# Three-Dimensional *In Vitro* Lymphangiogenesis Model in Tumor Microenvironment

**DOI:** 10.3389/fbioe.2021.697657

**Published:** 2021-10-04

**Authors:** Youngkyu Cho, Kyuhwan Na, Yesl Jun, Jihee Won, Ji Hun Yang, Seok Chung

**Affiliations:** ^1^ Department of IT Convergence, Korea University, Seoul, South Korea; ^2^ Samsung Research, Samsung Electronics Co. Ltd., Seoul, South Korea; ^3^ School of Mechanical Engineering, Korea University, Seoul, South Korea; ^4^ Departments of Pediatrics and Cellular & Molecular Medicine, Pediatric Diabetes Research Center, University of California, La Jolla, CA, United States; ^5^ Drug Discovery Platform Research Center, Therapeutics and Biotechnology Division, Korea Research Institute of Chemical Technology, Daejeon, South Korea; ^6^ Next&Bio Inc., Seoul, South Korea

**Keywords:** lymphangiogenesis, lymphatic vessel, interstitial flow, tumor microenvironment, organ-on-a-chip

## Abstract

Lymphangiogenesis is a stage of new lymphatic vessel formation in development and pathology, such as inflammation and tumor metastasis. Physiologically relevant models of lymphatic vessels have been in demand because studies on lymphatic vessels are required for understanding the mechanism of tumor metastasis. In this study, a new three-dimensional lymphangiogenesis model in a tumor microenvironment is proposed, using a newly designed macrofluidic platform. It is verified that controllable biochemical and biomechanical cues, which contribute to lymphangiogenesis, can be applied in this platform. In particular, this model demonstrates that a reconstituted lymphatic vessel has an *in vivo*–like lymphatic vessel in both physical and biochemical aspects. Since biomechanical stress with a biochemical factor influences robust directional lymphatic sprouting, whether our model closely approximates *in vivo*, the initial lymphatics in terms of the morphological and genetic signatures is investigated. Furthermore, attempting an incorporation with a tumor spheroid, this study successfully develops a complex tumor microenvironment model for use in lymphangiogenesis and reveals the microenvironment factors that contribute to tumor metastasis. As a first attempt at a coculture model, this reconstituted model is a novel system with a fully three-dimensional structure and can be a powerful tool for pathological drug screening or disease model.

## Introduction

The human circulatory system has two distinguished structures—the vascular and the lymphatic systems (Pugsley and Tabrizchi, 2000). The vascular system transports nutrients, gases, and hormones through blood vessels, and the lymphatic system which consists of lymphoid organs and lymphatic vessels helps in balancing tissue fluid, transporting immune cells, and draining connective tissue fluid (Alitalo, 2011; Noordergraaf, 2012). As the lymphatic vessels play an important role in immune functions and are spread throughout the body, some diseases, for example, inflammation and tumor metastasis, are influenced by overgrowth of lymphatic vessels or lymphedema in dysfunction of lymphatic vessels (Achen et al., 2005; Brouillard et al., 2014; Kim et al., 2014). Specifically, a robust tumor microenvironment facilitates new lymphatic sprouting and lymphangiogenesis due to the biochemical factors from tumors and peripheral stromal cells stimulating lymphatic endothelial cell migration and proliferation (Nagy et al., 2002; Chang et al., 2004; Tammela et al., 2005). The complex mechanism of lymphangiogenesis has been revealed in terms of genetic expression and biochemical cues using *in vivo* and *in vitro* models (Makinen et al., 2001), and a recent study pointed significant contribution of flow on lymphatic sprouting (Miteva et al., 2010). Leaky blood vasculature in tumor microenvironment generates interstitial flow and mechanical stress which induce lymphatic sprouting and thus lead to lymphangiogenesis (Kim et al., 2016; Choi et al., 2017). Lymphatic endothelial cells in the sprouts contribute intravasation of tumor cells into lymphatic vessels (Padera et al., 2002; Pisano et al., 2015). The complicated communication between lymphatic vessels and tumor cells has not been investigated more than a brief sketch because no advanced *in vitro* model has been proposed yet.

Previous *in vitro* models using conventional tools such as 2D cell culture dishes or an extracellular matrix (ECM)-coated plates cannot reflect the complex communication between lymphatic vessels and tumor cells because the cultured cells in the models locate without structural integrity and specific polarity, just exposed to culture medium in the one side and hard surface in the other (Leak and Jones, 1994; Shin et al., 2008; Kazenwadel et al., 2012). Coculturing lymphatic cells and cancer cells in transwell visualized invasion of cancer cells through lymphatic endothelium, however, without morphological relevance to *in vivo* tissues (Miteva et al., 2010; Pisano et al., 2015; Hikimoto et al., 2016; Triacca et al., 2017). A microfluidic system formed 3D lymphatic vessels and verified biochemical cues facilitating lymphatic sprouting but without physical interactions between cancer cells and lymphatic endothelium (Kim et al., 2016). Recently, biochemical effect between lymphatic endothelial cell and tumor was analyzed in 3D microfluidic co-culture system using colon cancer organoid, however, it is shown to need additional study related tumor metastasis (Frenkel et al., 2021).

This study proposes a new *in vitro* model for lymphangiogenesis in a tumor microenvironment, establishing tumor microenvironmental factors including tumor mass, ECM, and biochemical and fluidic components. A macroscale fluidic device (macrofluidic device) can easily incorporate a large amount of ECM and tumor mass in the channels and apply biochemical and biomechanical stimulation on the lymphatic endothelial cells. Combination of multiple microenvironmental factors presented synergic effect that ensured robust lymphangiogenesis. We finally demonstrated that biochemical and fluidic factors not only enhance the newly generated lymphatic sprouting but also have the potential to cause tumor metastasis.

## Materials and Methods

### Fabrication of Microfluidic Device

Soft lithography is used for fabricating poly-dimethylsiloxane (PDMS) devices. Channel patterned plastic mold was made by the injection molding process, and Sylgard 184 silicone solution with a curing agent (weight ratio, 10:1, Dow chemical, United States) was poured and cured on the plastic mold at 80°C for 1 h in a dry oven. The cured PDMS plate was detached from the plastic mold, and reservoirs of a medium channel and an ECM channel were punched using a 4- and 1-mm biopsy punch. The punched PDMS plate was used as a top part of the macrofluidic device and an unpunched PDMS plate was used for bottom. After autoclaving, the two parts were bonded by oxygen plasma treatment (CUTE; Femto Science, Korea). The ECM channel and medium channels are 2 × 1 mm and 4 × 1.2 mm in width and height, respectively (Supplementary Figure S1). Before ECM and cell filling, all channels were coated with 2 mg/ml of polydopamine solution for 2 h at room temperature and washed with distilled deionized water (DDW). The device was then dried for 5 h in an 80°C dry chamber to render the surface of the channel hydrophobic. A collagen type I (COL1) solution (Corning, USA) was mixed with 10 × PBS, 0.1 N NaOH, and DDW, and the final concentration of COL1 was adjusted at 2 mg/ml, pH 7.4. Well-mixed COL1 was filled into the ECM channel and incubated at 37°C in a 5% CO_2_ incubator to gel the COL1 solution. During the filling of COL1, edges of the ECM channel to cell culture channel prevented the COL1 solution from flowing out.

### Cell Culture and Tumor Spheroid Generation

Human dermal lymphatic endothelial cells (Lonza, Switzerland) were cultured in an endothelial basal medium 2 (EBM2, Lonza) supplemented by EGM2-MV BulletKit (Lonza) on a cell culture dish. For the cancer cells, MDA-MB-231, BT474, and A549 were obtained from the Korea cell line bank and cultured in RPMI 1640 (Lonza) containing 10% FBS on a cell culture dish. All cells were cultured at 37°C in a 5% CO_2_ incubator. To generate a tumor spheroid, a silicone concaved well (C-Well, Incyto, Korea) was attached to a 12-well plate and filled with 100% alcohol for rendering the surface from hydrophobic to hydrophilic, to minimize bubble formation during medium filling and cell seeding. After filling with alcohol, the well was washed with 1 × PBS three times. To prevent cell attachment, a 5% Pluronic F-12 was treated in a concave well for 30 min at room temperature and washed with 1 × PBS three times. 3 × 10^5^ cells/ml of a 2-ml cell suspension was filled into the concaved well, followed by culturing for 4 days at 37°C in a 5% CO_2_ incubator (Supplementary Figure S2).

### Cell Culture in a Macrofluidic Device

The lymphatic endothelial cells were detached using 0.25% trypsin/EDTA (Thermo Fisher Scientific, USA) and prepared at a density of 5 × 10^5^ cells/ml. The cell culturing channels were filled with 100 μl of a lymphatic endothelial cell suspension, and the devices were placed vertically in the 5% CO_2_ incubator for 1 h at 37°C to attach the lymphatic endothelial cells to the COL1 hydrogel. After cell attachment, a cell culture medium was refreshed in all medium channels. The lymphatic endothelial cell was cultured for 3 days to cover the cell culture channels, and the medium was refreshed daily. To stimulate the lymphatic cells, 100 ng/ml of VEGF-A and VEGF-C (PeproTech, USA) were filled in the lymphangiogenic factor channel, and the height difference of the medium in each medium channel was maintained to apply interstitial flow. Flow calculation by Darcy’s law is described in supplementary information. To coculture with invasive cancer cells, 100 μl of 1 × 10^6^ cells/ml MDA-MB-231 were filled in the lymphangiogenic channel after lymphangiogenesis (Supplementary Figure S3). In the case of a coculture with a tumor spheroid, spheroids made in microwell were first collected by pipetting and mixed with the COL1 solution, and then the mixture was filled in the ECM channel and incubated at 37°C in the 5% CO_2_ incubator for 30 min.

### Molecular Distribution and Computational Simulation

The growth factor gradient in the macrofluidic device was confirmed by experiment and computational simulation. 10 μM of 40 kDa FITC-dextran and 10 kDa RITC-dextran were filled into the lymphangiogenic factor channel. Dextran diffusion was monitored using a fluorescence microscope (Axio Observer D1; Carl Zeiss, Germany) every 3 h, and the intensity profile was analyzed using ImageJ (NIH). In the computational simulation, the molecular transport was simulated using a COMSOL Multiphysics 5.4 (COMSOL, Sweden). Coefficients applied in the simulation are listed as supplementary information (Supplementary Figure S4 and Supplementary Table S1).

### Immunofluorescence Staining and Morphological Analysis

Immunofluorescence and phase-contrast images were used for analyzing the lymphatic vessels. For immunofluorescence staining, a macrofluidic device after experiments was fixed with 4% paraformaldehyde and incubated for 20 min at room temperature. Fixed cells were permeabilized using 0.1% Triton X-100 for 10 min. To prevent nonspecific binding, the samples were blocked with 1% BSA and incubated for 1 h at room temperature. Primary antibodies with 1% BSA, anti–VE-cadherin, anti-laminin, and anti-VEGFR3 (all from Abcam, United Kingdom) were filled into the channels and incubated for 2 h. After washing with 1x PBS three times, a mixture of Alexa Fluor conjugated goat anti-rabbit secondary antibody, rhodamine-phalloidin, and 4′,6-diamidino-2-phenylindole (all from Thermo Fisher Scientific) was filled in the device and incubated for 2 h. Images were obtained using a fluorescence microscope and a confocal microscope (LSM700; Carl Zeiss). A morphological analysis was conducted using ImageJ.

### Quantitative mRNA Expression Analysis

After experiments, cells were collected using 0.25% trypsin/EDTA, and the total RNA was acquired using an RNeasy Mini Kit (Qiagen, USA). The RNA concentration was measured using a Nanodrop spectrometer (Thermo Fisher Scientific), and cDNA was synthesized using high-capacity RNA-to-cDNA (Applied Biosystems, United States). A mixture of synthesized cDNA, target gene primers, and a QuantiTect SYBR Green PCR kit was filled into the PCR tubes, and qRT-PCR was applied using a StepOne Real-Time PCR system (Applied Biosystems). Gene expression was normalized by a housekeeping gene, GAPDH, and the relative gene expression was calculated using the comparative Ct method. The designed target gene primers are described (Table S2).

### Cytokine Analysis

Cancer-attractive CCL21 was quantified using commercial enzyme-linked immunosorbent assay (ELISA) kits (Abcam). The medium was collected in a lymphangiogenic factor channel and a stimulated channel, and ELISA was applied using protocols provided by the manufacturer. The optical intensity in each well was read using a microplate spectrometer by measuring the absorbance at 450 nm, and a standard curve was obtained using four-parameter logistic regression.

### Statistical Analysis

Quantified experimental data were expressed as the mean standard error, and the statistical significance was determined using an unpaired two-tail Student’s *t*-test. The significance was considered based on*p < 0.05, **p < 0.01, ***p < 0.001, and ****p < 0.0001.

## Results

### Macrofluidic Device for Lymphangiogenesis

**FIGURE 1:**
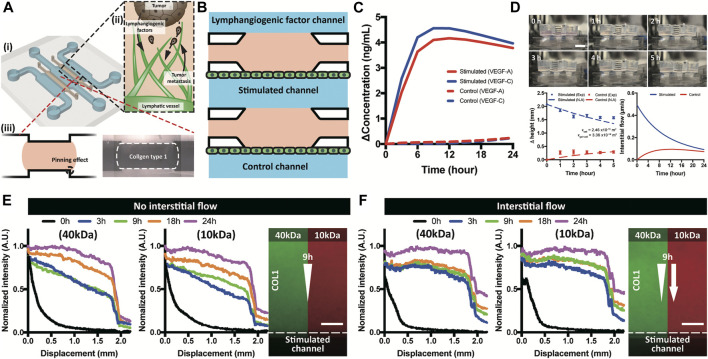
Brief illustration of a macrofluidic device and molecular distribution. **(A)** ECM incorporated macrofluidic device and illustration of lymphangiogenesis in a tumor microenvironment. **(B)** Top view of the macrofluidic device. **(C)** Generated growth factor gradient on the lymphatic endothelium in a stimulated and control channel. **(D)** Interstitial flow generation using hydraulic head difference and a numerical analysis. **(E,F)** Experimental confirmation of molecular distribution in COL1 under absence and applied interstitial flow. Scale bar is 200 μm.

3D lymphangiogenesis into ECM by growth factors and interstitial flow was successfully reconstituted in a macrofluidic device with tumor spheroids. Step-edge around the ECM channel helped the COL1 hydrogel to be incorporated only in the ECM channel by a pinning effect (Figure 1Ai). Meniscus pressure (P_meniscus_) to prevent COL1 hydrogel bursting was calculated using interfacial energy equation, and filling pressure (P_filling_) of the COL1 hydrogel was derived using the Poiseuille equation (Supplementary Figure S5). The two equations could successfully predict a stable COL1 hydrogel filling condition, which can confirm stable incorporation of the COL1 hydrogel regardless of COL1 filling velocity. Chemical and physical cues were applied in the lymphangiogenic factor channel with lymphatic endothelial cells cultured on the stimulated and control channels (Figure 1B).

Chemical gradient near the lymphatic endothelium verified by computational simulation showed that the only lymphatic endothelium in the stimulated channel was affected by the gradient of growth factors (VEGF-A and -C) (Figure 1C and Supplementary Figure S6). The three channel layout of lymphangiogenic factor–stimulated control channels enabled the simulating group and the control group to be tested in just one device at the same time. Gradient formed in the device was confirmed using 40 and 10 kDa dextran representing VEGF-A and -C, respectively. The applied dextran presented two phases of distribution, linear in the COL1 hydrogel and steep decrease over the lymphatic endothelium (Figure 1E and Supplementary Figure S8A,B). Interstitial flow generated by hydraulic head difference was calculated using Darcy’s law with assumption of the COL1 hydrogel as a porous structure (Supplementary Figure S7) (Cross et al., 2010; Shin et al., 2012). Darcy’s permeabilities of the COL1 hydrogel (1.04 × 10^−13^ m^2^) and the lymphatic endothelium (2.46 × 10^−16^ m^2^), interstitial flow (in range of 0.49 to 0.09 μm/s), and the Peclet number (in the range from 3 to 15 for 24 h) were calculated (Figure 1D and Supplementary Figure S7). Note the flat gradient due to the convective transport in the COL1 hydrogel and step decrease over the lymphatic endothelium (Figure 1F and Supplementary Figure S8).

### Synergic Regulation of Lymphangiogenesis by Growth Factors

**FIGURE 2:**
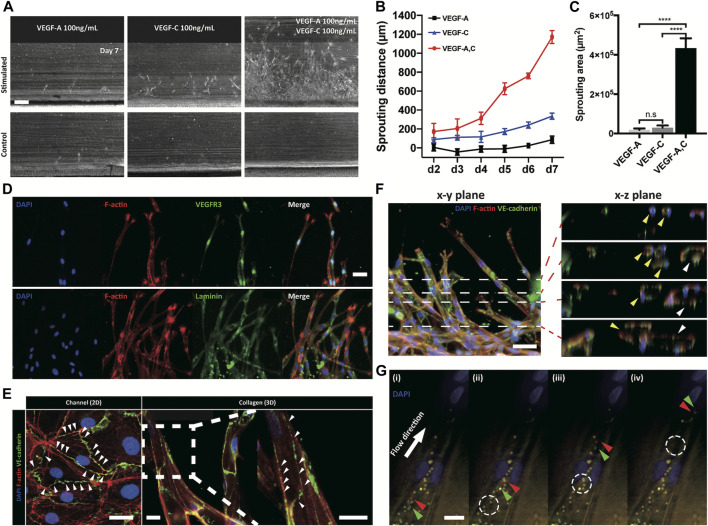
Response of lymphatic sprouting under growth factor stimulation and characterization of generated lymphatic vessel. **(A)** Lymphatic sprouting under three conditions, **(B)** quantified sprouting distance, and **(C)** area in COL1. Scale bar indicates 200 μm (*n* = 4 and error bar indicates standard deviation. *** and **** indicate *p* < 0.001 and *p* < 0.0001, respectively). **(D)** Lymphatic vessel expressing VEGFR3 and laminin, which indicate a lymphatic specific marker and a basement membrane (scale bar 50 μm). **(E)** Button-like junctional expression of a lymphatic endothelial cell and a sprouted lymphatic vessel. White arrowhead indicates discontinuous point of the junction. Scale bars indicate 30 and 100 μm in the channel (2D) and collagen (3D), respectively. **(F, G)** Luminal structure of sprouted lymphatic vessel using confocal microscopy. White dashed lines in the x-y plane indicate sequential images in the x-z plane, and each colored arrowhead indicates one sprouted vessel (yellow and white). Scale bar is 50 μm. **(G)** Sequential images of moving microparticles in the sprouting lymphatic vessel. Green and red arrowheads indicate moving microparticles, and white dashed circle indicates previous position of moving particles.

VEGF families were known to regulate lymphangiogenesis with VEGFR3 on lymphatic endothelial cells (Alitalo and Detmar, 2012). We monitored lymphatic sprouting under the gradients of VEGF-A, VEGF-C, and their combination (Figure 2A and Supplementary Figure S9). VEGF-C gradient induced lymphatic sprouting, while VEGF-A gradient did not. Interestingly, combined gradient of VEGF-A and C dramatically facilitated lymphatic sprouting (Figure 2B) with increased vessel density (Figure 2C), proving dramatic synergetic effect of the combined gradient of VEGF families on the 3D lymphangiogenesis into the COL1 hydrogel. The sprouted lymphatic endothelial cells strongly expressed a specific VEGF receptor, VEGFR3, and remodeled basement membrane by laminin around them (Figure 2D). Newly generated 3D initial lymphatic vessels had a distinguished button-like cell–cell junctional expression for easy draining of interstitial fluid (Baluk et al., 2007; Yao et al., 2012) (Figure 2E). Cross-sectional images showed circular 3D lumen structures with multiple connected vessels (Figure 2F). 2 μm microparticles applied in the cell-cultured channels flowed into the lymphatic vessels without leakage (Figure 2G). 3D reconstitution of physiologically relevant lymphatic vessels in the COL1 hydrogel by the combined application of growth factors was confirmed by biological expression and the physical structure.

### Synergic Regulation of Lymphangiogenesis by Interstitial Flow and Growth Factor

**FIGURE 3:**
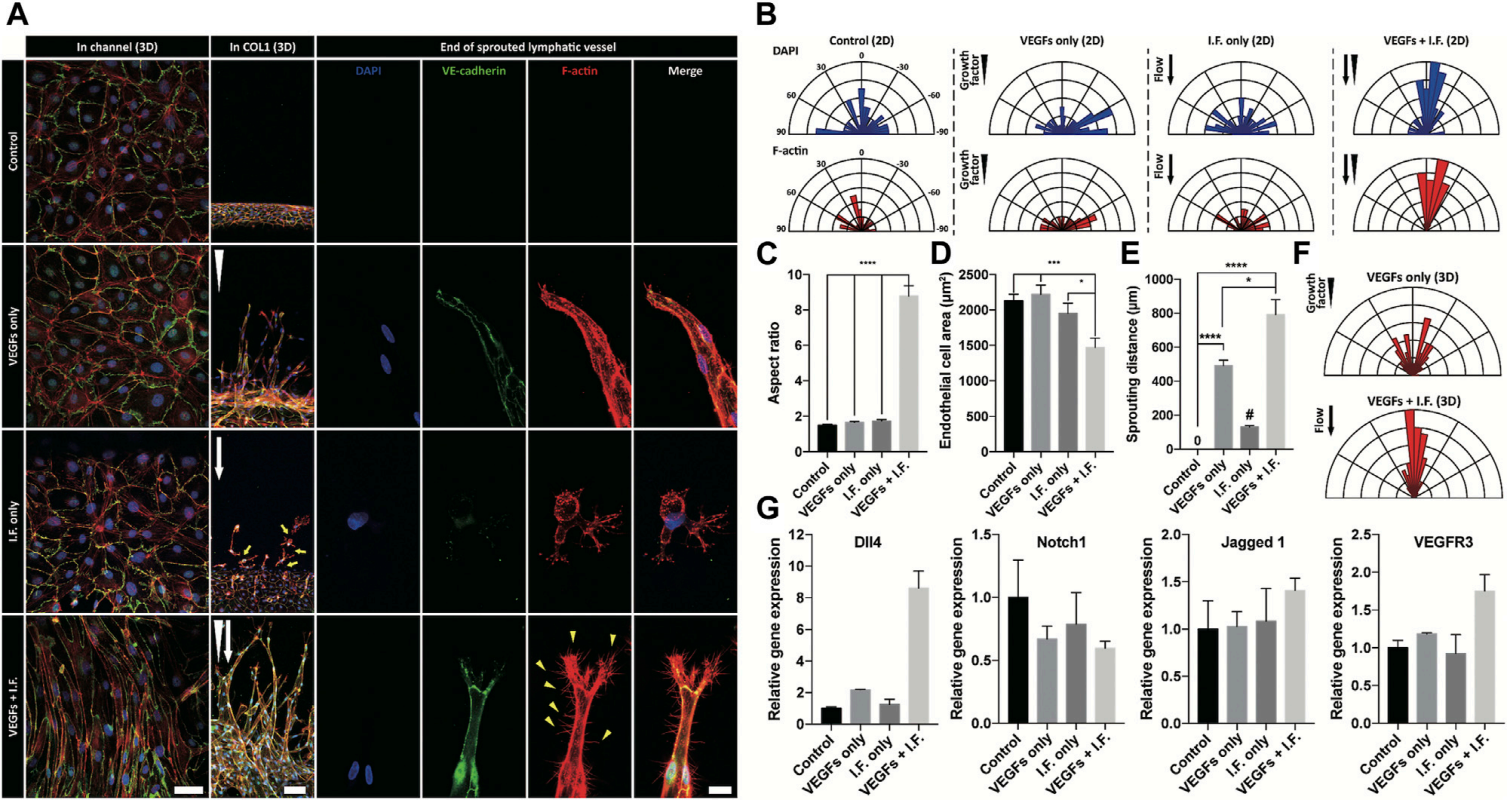
Morphological analysis of lymphatic endothelial cells and sprouted lymphatic vessel under growth factor and interstitial flow stimulation. **(A)** Immunofluorescent images of lymphatic endothelial cells in a cell-cultured channel **(first column)**, a sprouted lymphatic vessel in COL1 **(middle column)**, and end of sprouted lymphatic vessel **(third column)**. White arrowhead and arrow indicate growth factor gradient and interstitial flow direction, respectively. Scale bars are 50 μm **(first column)**, 100 μm **(second column)**, and 20 μm (third column). **(B)** Polar distribution of lymphatic endothelial cell’s orientation in the cell-cultured channel (2D) in terms of nuclei **(top row)** and cell body **(bottom row)**. **(C, D)** Morphological analysis of lymphatic endothelial cell in the cell-cultured channel in terms of **(C)** aspect ratio and **(D)** cell area (error bar indicates standard error, and *, ***, and **** indicate *p* < 0.05, *p* < 0.001 and *p* < 0.0001, respectively). **(E)** Quantification of lymphatic sprouting distance in COL1 under growth factor and interstitial flow stimulation. # indicates single cell migration (error bar indicates standard error, and *and **** indicate *p* < 0.05, *p* < 0.001, and *p* < 0.0001). **(F)** Polar distribution of sprouted lymphatic vessel’s direction in COL1. **(G)** Gene-level expression related to tip cell and sprouting lymphatic vessel (Dll4, Notch1, Jagged 1, and VEGFR3).

Shear stress is well known to lead morphological change of endothelial cells, however not of lymphatic endothelial cells (Ye et al., 2014; Poduri et al., 2017). In our experiments, under the application of interstitial flow or growth factors only, lymphatic endothelial cells in the cell-cultured channel showed only random alignment. However co-stimulation of interstitial flow and growth factors dramatically changed the morphology of the cultured lymphatic endothelial cells (first column in Figure 3A) and induced active lymphangiogenesis into the COL1 hydrogel (Figures 3A,E). The co-stimulation was proved to make the lymphatic endothelial cell elongate toward the flow direction (Figure 3 B), with an increased aspect ratio (8.79), decreased circularity (0.19) (Figure 3C), and decreased cell area (Figure 3D). These results showed the dramatic synergic effect of the interstitial flow and growth factors on the 2D alignment of lymphatic endothelial cells and 3D sprouting into the COL1 hydrogel (Figure 3E). The co-stimulation not only enhanced but also guided lymphatic sprouting into the COL1 hydrogel (Figure 3F). Note robustly facilitated filopodial protrusion into the COL1 hydrogel by the synergic effect of the interstitial flow and the growth factor. Strong upregulation of Dll4 could answer for the synergic effect (Figure 3G). However, different from studies, notch signaling activation by high Dll4 expression was not noticed, which could be explained by upregulated Jagged 1 expression as an antagonist in notch signaling (Benedito et al., 2009). VEGFR3 locating at notch 1 downstream increased under the synergic condition, suggesting mechanotransduction responses in lymphatic endothelial cells.

### Tumor Invasion Toward Lymphatic Vessels

**FIGURE 4:**
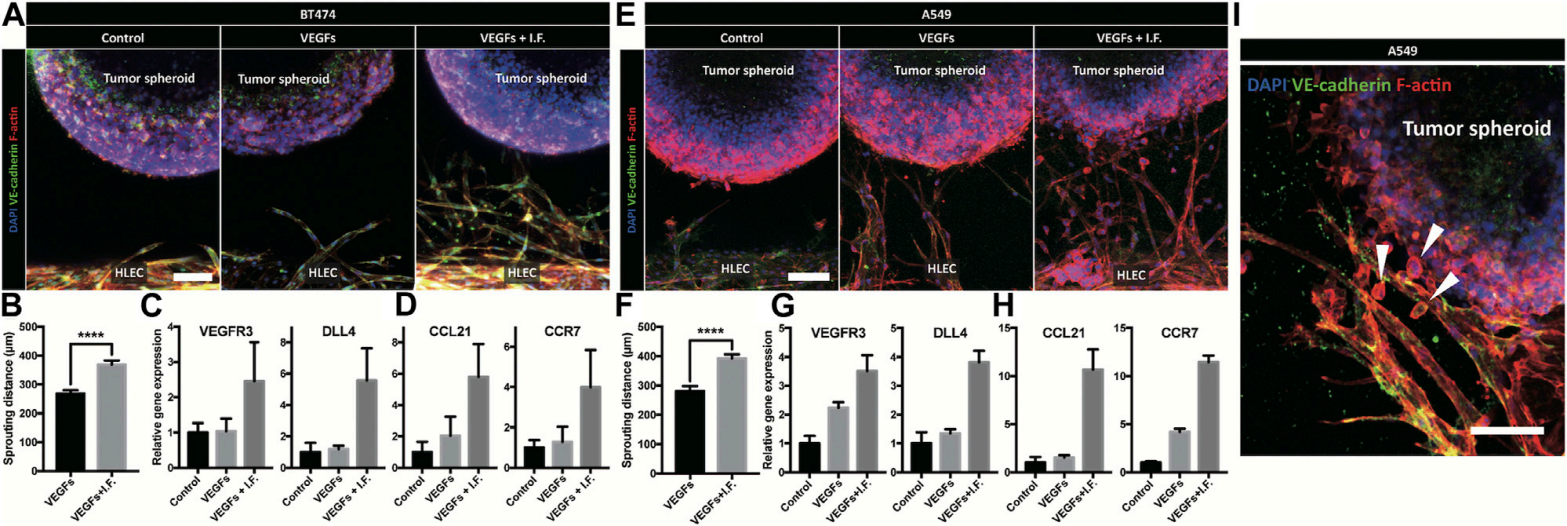
Coculture model of tumor spheroid and lymphatic endothelial cell under a noninvasive cancer spheroid and a very low invasive cancer spheroid. **(A)** Immunofluorescent micrograph of the lymphatic vessel with a noninvasive cancer spheroid (BT474). Scale bar is 100 μm. **(B)** Quantification of lymphatic sprouting toward tumor spheroid (BT474 and **** indicates *p* < 0.0001). **(C, D**) Gene level expression related to **(C)** lymphatic sprouting and **(D)** tumor metastasis in BT474. **(E)** Immunofluorescent micrograph of the lymphatic vessel with a very low-invasive cancer spheroid (A549). Scale bar is 100 μm. **(F)** Quantification of lymphatic sprouting toward a tumor spheroid (A549 and **** indicates *p* < 0.0001). **(G, H)** Gene-level expression related to **(G)** lymphatic sprouting and **(H)** tumor metastasis in A549. **(I)** Cancer invasion from tumor spheroid to peritumoral lymphatic vessel under interstitial flow and growth factor stimulation. Scale bar is 100 μm. White arrowheads indicate a migrated cancer cell.

**FIGURE 5:**
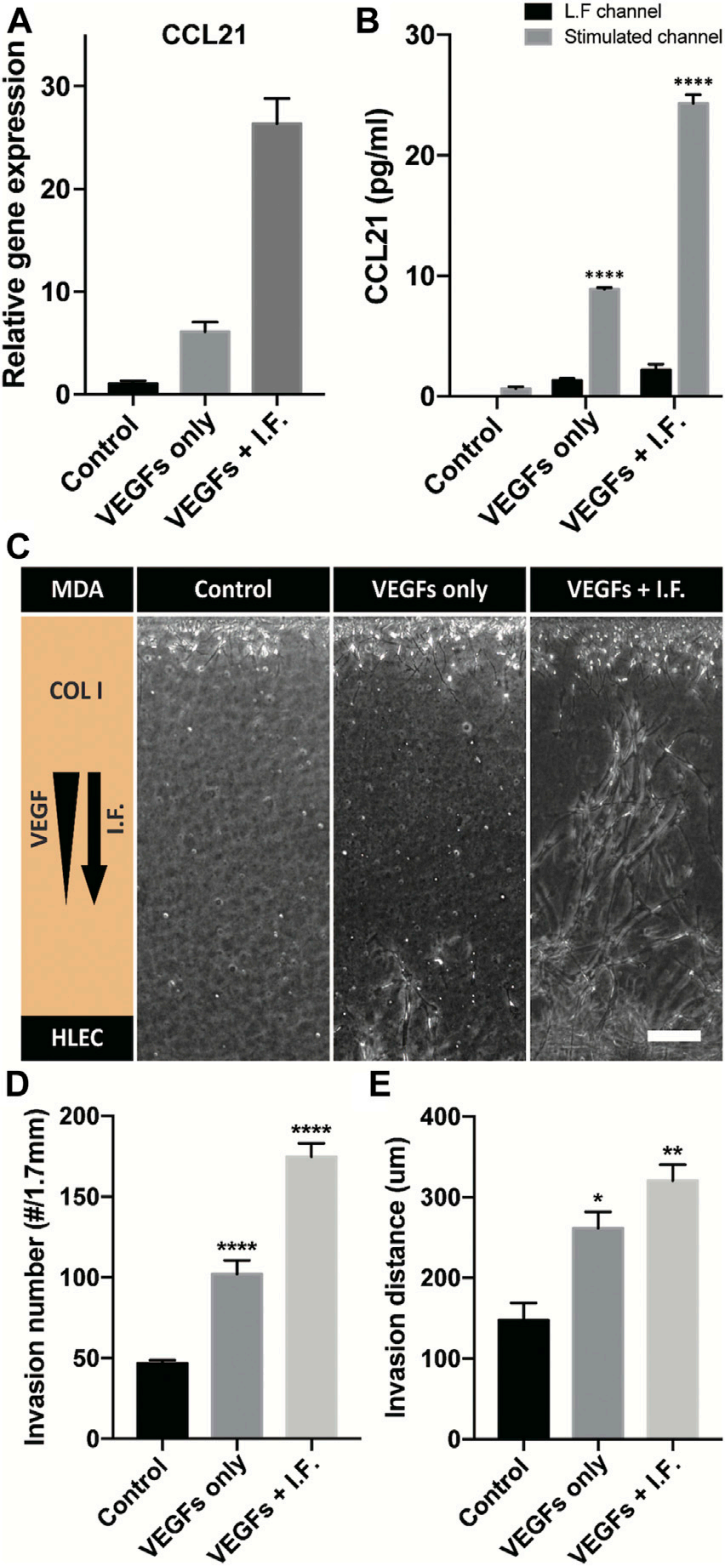
CCL21 expression and coculture model of a high-invasive cancer cell and a lymphatic endothelial cell. **(A)** Gene-level expression of CCL21 on lymphatic endothelial cells under lymphangiogenic factors. **(B)** CCL21 expression using ELISA in a cell-cultured channel and the lymphangiogenic factor channel (**** indicates *p* < 0.0001, and CCL21 expressions are compared with control in L.F. channel). **(C)** Micrograph of lymphatic sprouting and cancer cell invasion under lymphangiogenic factors. Scale bar is 200 μm. **(D, E)** Quantification of cancer invasion in terms of **(D)** invaded cell number and **(E)** invasion distance. The results are compared with control (*, **, and **** indicate *p* < 0.05, *p* < 0.001, and *p* < 0.0001).

Lymphatic vessels form the tumor microenvironment with growth factors secreted by cancer cells and interstitial flow from leaky blood vessels at the tumor site. A tumor spheroid was adapted in the macrofluidic device and cocultured with lymphatic endothelial cells (Figure 1Ai). The tumor spheroid was acquired from a cancer cell aggregate in a micro concave well, with two noninvasive breast cancer cell lines, BT474 and A549 (Supplementary Figure S10A). They were 400 μm in diameter, with a necrotic core 200 μm in diameter (Supplementary Figure S10B). The spheroids embedded in the macrofluidic device in COL1 similarly grew under all culture conditions (Supplementary Figure S10C,D). Note the cultured spheroid and active lymphangiogenic response in the synergic culture condition of VEGFs and interstitial flow (Figures 4A–H). CCL21 was upregulated in lymphangiogenic sprouts, possibly recruiting CCR7 expressing cancer cells toward the lymphatic vessels. Note CCR7 upregulation in cancer cells. Despite there being no visible clue for the enhanced tumor invasion, mRNA expression showed a potential for tumor metastasis of the synergic stimulation of growth factors and interstitial flow, correlating well with CCL21-CCR7 as the main mechanism of tumor metastasis by lymphatic endothelial cells (Lanati et al., 2010). Interestingly, A549 spheroid showed escaping and shedding of cells in the immediate vicinity of lymphatic vessels (Figure4I). Investigation using a highly invasive breast cancer cell line, MDA-MB-231, also showed increased expression of CCL21 in the synergic stimulation (Figure5A). Its protein level was well matched with the gene expressing pattern (Figure 5B), but interestingly, CCL21 concentration was slightly increased in the lymphangiogenic factor channel (LF channel) with lymphatic endothelial cells. CCL21 molecules seemed to transport against the interstitial flow from the LF channel, which was simulated considering the Peclét number of the molecule (Supplementary Figure S11). Note the active lymphatic sprouting under the synergic stimuli and enhanced invasion of MDA-MB-231 cells toward the lymphatic vessels (Figures 5C–E).

## Discussion

When a tumor grows, the microenvironment including the blood vessels, lymphatic vessels, stromal cells, and ECM is altered by the communication of each component and supports the tumor growth, invasion, and metastasis. Growth factors, which are secreted from the tumor or stromal cell such as the VEGF family, facilitate angiogenesis and an expansion of the leaky vasculature, increasing the interstitial fluid pressure (Jain, 2005; Carmeliet and Jain, 2011). In addition to angiogenesis, lymphangiogenesis, a new lymphatic vessel generation, occurs in the peripheral site of the tumor sharing angiogenic factors with blood vessels. The interstitial flow from leaky blood vasculatures was known to act as a guide achieving robust generation of lymphangiogenesis (Dafni et al., 2002; Kim et al., 2016; Choi et al., 2017). Similar to the blood vessels, recruited lymphatic vessels regulate metastasis progress by cancer cell invasion toward the lymph node (Issa et al., 2009; Christiansen and Detmar, 2011). This study demonstrated the synergic effect of growth factors and interstitial flow on the lymphatic sprouting and genetic expression, using a newly developed macrofluidic device which can easily modulate the biochemical and mechanical stimulation to macroscale tissues. The macrofluidic device formed physiologically relevant lymphangiogenesis model near the tumor site under precisely integrated biochemical and mechanical stimulation.

VEGF-C has been known as a dominant regulator for lymphangiogenesis, binding VEGF3 on lymphatic endothelial cells. VEFG-A has also been reported as another regulator for not only angiogenesis but also lymphangiogenesis by maintaining VEGF-C/VEGFR signaling (Hirakawa et al., 2003; Hirakawa et al., 2005). Our important finding is synergic effect of VEGF-A and -C gradients over the weak 3D lymphangiogenic sprouting into the COL1 hydrogel under VEGF-A gradient. Physiological relevance of the reconstituted 3D lymphatic vessels to the *in vivo* structure was confirmed by confocal microscope images, unique button-like junctions which never been reported in previous *in vitro* models, and hollow tunnels inside enabling transporting microparticles, draining of connective fluid and homing immune cells (Baluk et al., 2007; Yao et al., 2012). Another interesting finding is additional synergic effect of VEGF gradient and interstitial flow on lymphangiogenesis. Lymphatic endothelial cells actively change their morphology by elongating to the direction of flow and gradient. Shear stress, a strong regulator on the blood endothelial cells (Ye et al., 2014; Poduri et al., 2017), could not induce morphological change of the lymphatic endothelial cells. The synergic effect of biochemical growth factors and mechanical stress upregulated VEGFR3 expression, which did not precisely correlate with previous reports suggesting a mechanosensory role of VEGFR3 in lymphatic endothelial cells (Coon et al., 2015). Different from endothelial cells (Kume, 2009), lymphatic endothelial cells presented Dll4 upregulation through VEGF signaling but without notch activation due to the upregulated Jagged 1 under the synergic stimulation (Suchting and Eichmann, 2009; Zhang et al., 2018).

The macrofluidic device demonstrated communication between the lymphatic vessels and tumor spheroids mediated by CCL21–CCR7 axis. During tumor metastasis through the initial lymphatics, lymphatic endothelial cells were known to secrete CCL21 and attracted cancer cells expressing CCR7 and CCL21 receptors (Wiley et al., 2001; Shields et al., 2007). Some studies showed that microenvironmental factors of the tumor increase the CCL21 expression level in the lymphatic endothelial cells, and induced a tumor invasion (Miteva et al., 2010; Pisano et al., 2015). In the macrofluidic device, the CCL21 level in the lymphatic endothelial cells was facilitated by coculture with noninvasive tumor spheroids (BT474 and A549) under the synergic stimuli. Clue for the enhanced cancer invasion of noninvasive tumor cells by the synergic stimuli into the peritumoral lymphatic vessel was also verified. Interestingly, we found the upregulation of CCR7 in the noninvasive cancer spheroids under the synergic stimulation. Highly invasive cancer cell line (MDA-MB-231) presented the similar enhanced invasion following CCL21 expression, indirectly confirming the commonality of the CCL21-CCR7 axis on the enhanced caner malignancy under the tumor microenvironment. We believe that the macrofluidic device can present a physiologically relevant model of lymphangiogenesis in a tumor microenvironment regarding the morphological and genetic expression and suggest a new tool for research on lymphangiogenesis in a pathology or targeted therapy. This platform can be used to drug testing for anti-lymphangiogenesis by applying anti-VEGF antibody and VEGFR3 blocking. These two types of drugs will verify suppression of lymphangiogenesis about biochemical stimulation and, especially, blocking VEGFR3 can be tested for inhibition of mechanical stimulation such as interstitial flow. Also, since CCL21–CCR7 axis mediates tumor metastasis, blocking CCR7 in cancer cell or inhibition CCL21 secretion can test for restrict metastasis.

## Data Availability

The original contributions presented in the study are included in the article/Supplementary Material; further inquiries can be directed to the corresponding author.
