# Supplementary material for: Three-Dimensional In Vitro Lymphangiogenesis Model in Tumor Microenvironment
Source: Front Bioeng Biotechnol. 2021 Oct 4;9:697657. doi: 10.3389/fbioe.2021.697657 (PMC8520924; doi:10.3389/fbioe.2021.697657)
Supplement: Supplementary file 1 [file DataSheet1.docx]

Supplementary Material

Three-dimensional in vitro lymphangiogenesis model in tumor microenvironment

Youngkyu Cho^1,4^, Kyuhwan Na^2^, Yesl Jun^3,6^, Jihee Won^2^, Ji Hun Yang^2,5^*, Seok Chung^1,2^*


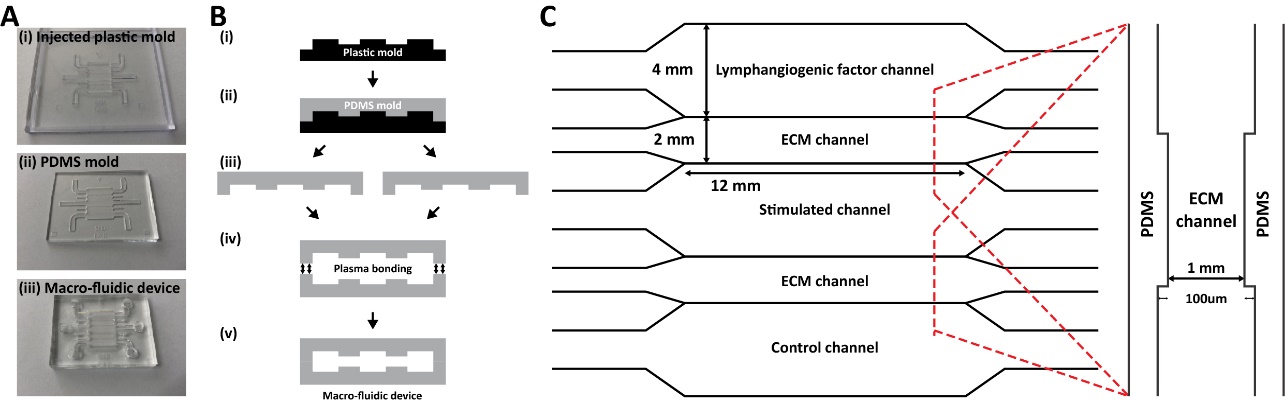


**Figure S1**. Illustration of macro fluidic platform. (a) Real images of (i) plastic injected mold, (ii) baked PDMS mold, and (iii) fully fabricated fluidic device. (b) Fabrication process of macro fluidic platform. (c) Schematic illustration of a macro fluidic platform in top view (left) and cross-sectional view (right).

**
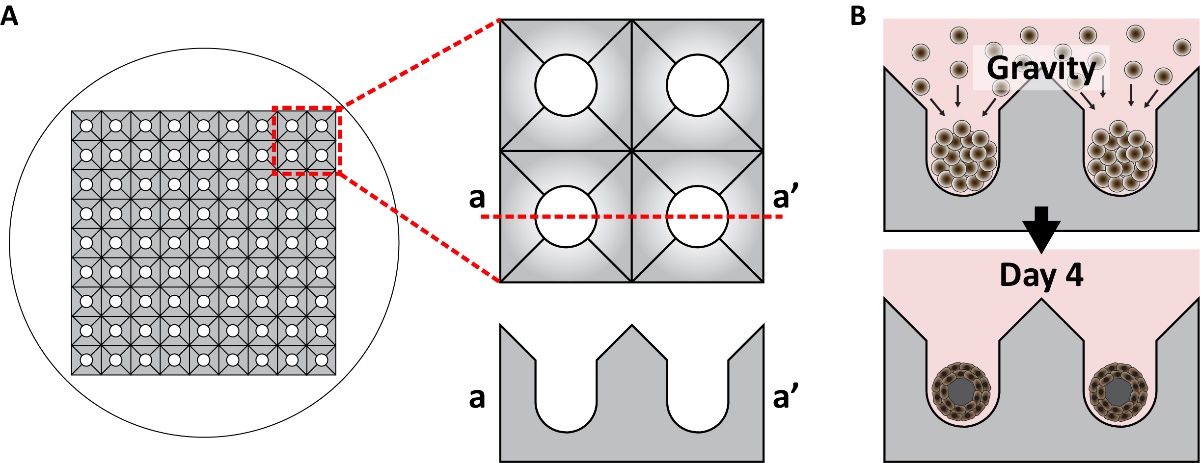
**

**Figure S2**. (a) Illustration of concaved well for generating tumor spheroid. Red dashed line indicates magnified image of concaved well and red dashed line (a-a’) indicates cross-sectional image. (b) Cell seeding and culturing process in concaved well. Cell suspension seeded on concaved well and paused for 20 min before entering concaved well. The tumor spheroid generated for 4 days.


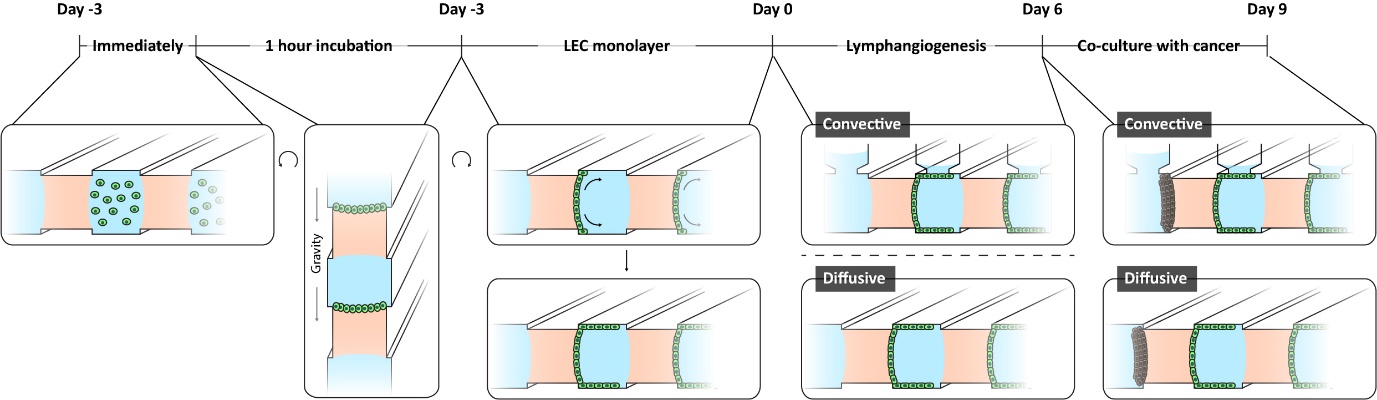


**Figure S3**. Brief illustration of cell seeding and culturing in the meso-fluidic device. Gravity pressure generated lymphatic endothelial cell attachment on COL1. Cell cultured during 3 days in the channels for covering cell culture channel. The medium contained lymphangiogenic factors filled in lymphangiogenic channel. Co-culturing with cancer cells, cancer cells were seeded in lymphangiogenic channel.


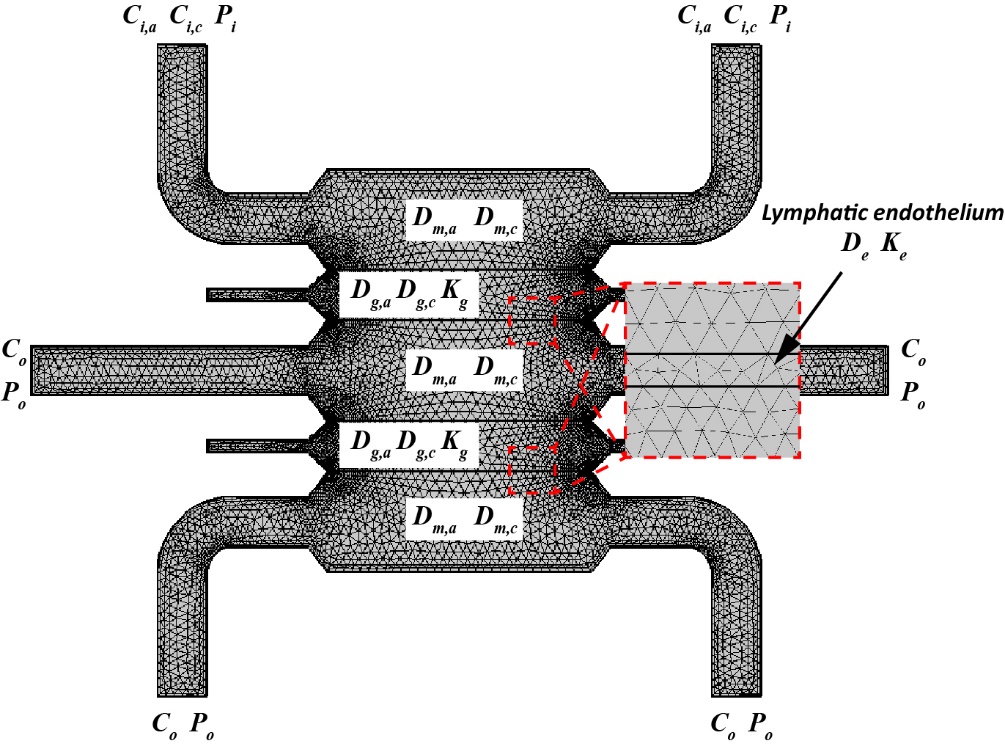


**Figure S4**. Mesh illustration and coefficient notation for computational simulation. Enlarged image (red dashed box) indicates lymphatic endothelium.

**Table S1**. Numerical values for computational simulation

| Description | Notation | Value | Dimension | Ref. |
| --- | --- | --- | --- | --- |
| Initial concentration of VEGF-A | *C_i,a_* | 2.5 × 10^-6^ | mol/m^3^ | Experimental value |
| Initial concentration of VEGF-C | *C_i,c_* | 5 × 10^-6^ | mol/m^3^ | Experimental value |
| Outlet of VEGF concentration | *C_o_* | 0 | mol/m^3^ | Experimental value |
| Diffusion coefficient of VEGF-A at medium | *D_m,a_* | 6.94 × 10^-11^ | m^2^/s | [1] |
| Diffusion coefficient of VEGF-C at medium | *D_m,c_* | 6.11 × 10^-11^ | m^2^/s | [1] |
| Diffusion coefficient of VEGF-A at COL1 | *D_g,a_* | 5.8 × 10^-11^ | m^2^/s | [2] |
| Diffusion coefficient of VEGF-C at COL1 | *D_g,c_* | 6.6 × 10^-11^ | m^2^/s | [3] |
| Diffusion coefficient of lymphatic endothelium | *D_e_* | 1.57 × 10^-12^ | m^2^/s | [4] |
| Inlet pressure | *P_i_* | 8 | Pa | Experimental value |
| Outlet pressure | *P_o_* | 0 | Pa | Experimental value |
| Medium density | *ρ* | 993.37 | kg/m^3^ | Experimental value |
| Dynamic viscosity | *μ* | 0.6913 | Pa⋅s | Experimental value |
| Permeability of COL1 | *K_g_* | 1.04 × 10^-13^ | m^2^ | Obtained by experiment |
| Permeability of lymphatic endothelium | *K_e_* | 2.46 × 10^-16^ | m^2^ | Obtained by experiment |

**Table S2**. Primer design for qRT-PCR

| Target gene | | Primer sequence | Tm (°C) |
| --- | --- | --- | --- |
| VEGFR3 | Forward | GAGTTCCTGGCTTCCCGAAA | 59.0 |
|  | Reverse | AGATGCTTTCAGGGGCCATC | 59.1 |
| PROX-1 | Forward | GAGATGTGCGAGCTAGACCC | 59.0 |
|  | Reverse | CACAGTGTCCACAACTTGCG | 59.0 |
| Dll4 | Forward | CCACTTCGGCCACTATGTGT | 59.1 |
|  | Reverse | GTACATTGCCAGGGAGTGCT | 59.1 |
| Notch1 | Forward | CAACTGCCAGAACCTTGTGC | 59.0 |
|  | Reverse | GGCAACGTCAACACCTTGTC | 59.0 |
| Jagged-1 | Forward | CTACAACCGTGCCAGTGACT | 59.0 |
|  | Reverse | CTGACTCTTGCACTTCCCGT | 59.0 |
| CCL21 | Forward | TACCGGAAGCAGGAACCAAG | 58.7 |
|  | Reverse | TACCGGAAGCAGGAACCAAG | 59.0 |
| CXCL12 | Forward | AGCCAACGTCAAGCATCTCA | 59.0 |
|  | Reverse | TCGGGTCAATGCACACTTGT | 59.2 |
| CCR7 | Forward | ATCAGTCTGGACCGCTACCT | 59.0 |
|  | Reverse | ATCTGCCTCACTGACGTTGG | 59.1 |
| CXCR4 | Forward | ATCAGTCTGGACCGCTACCT | 59.0 |
|  | Reverse | ATCTGCCTCACTGACGTTGG | 59.1 |
| GAPDH | Forward | TCCAGAACATCATCCCTGCC | 58.5 |
|  | Reverse | GCCTGCTTCACCACCTTCTT | 59.6 |


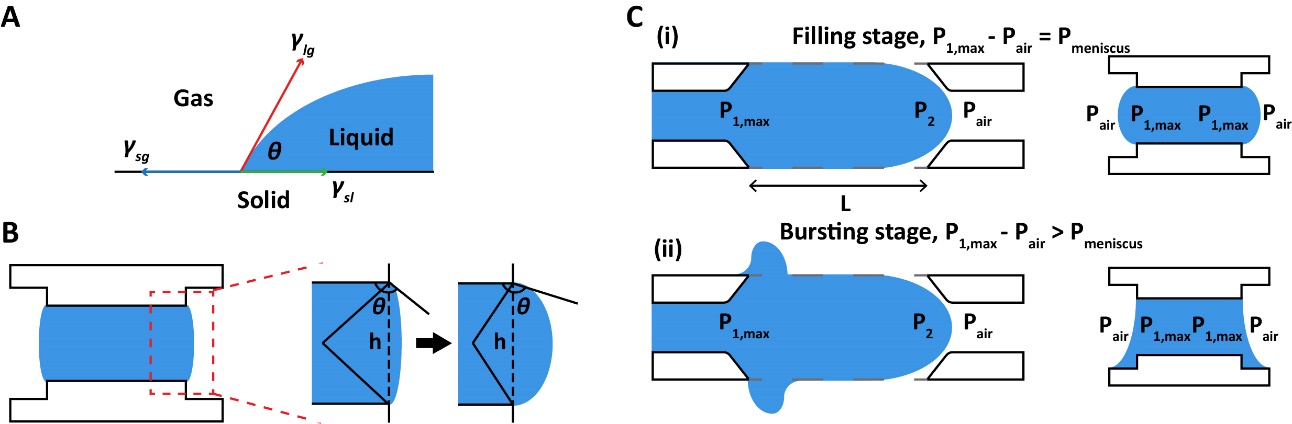


**Figure S5.** (a) Illustration of gas-liquid-solid interface energy equilibrium. (b) Volume change illustration of liquid in ECM channel, filling liquid. (c) (i) Illustration of liquid pressure in filling stage at top view (left) and cross-sectional view (right). (ii) Bursting stage illustration of liquid pressure at top view (left) and cross-sectional view (right).

For incorporating COL1 in the fluidic device, the COL1 solution filling process was described based on energy changes of liquid-solid–gas interface **(Fig. 12(a))**. The governing equation of total interface energy (**Eq. 1**) is

$U_{t}=\gamma_{lg}A_{lg}+\gamma_{sl}A_{sl}+\gamma_{sg}A_{sg}$ (1)

where *A_lg_*, *A_sl_*, and *A_sg_* are liquid–gas, solid–liquid, and solid–gas interface areas, and *γ_lg_*, *γ_sl_*, and *γ_sg_* are liquid**-**gas, solid–liquid, and solid–gas surface tensions ^5,6^. The surface tension has equilibrium related with contact angle *θ* based on Young’s equation (**Eq. 2**), and the expression of energy (**Eq. 3**) can be reduced by Eq. (2) in (1).

$\gamma_{sg}=\gamma_{lg}\cos\theta+\gamma_{sl}$ (2)

$U_{t}=U_{0}-\gamma_{lg}\left( A_{sl}\cos\theta-A_{lg} \right)$ (3)

Liquid pressure at the meniscus is derived as the change in total interface energy by the liquid volume change (**Eq. 4**), and the volume of liquid can be calculated in Eq. (5).

$\Delta P=\frac{dU_{t}}{dV_{l}}=-\gamma_{lg}\left( \frac{{dA}_{sl}}{dV_{l}}\cos\theta-\frac{{dA}_{lg}}{dV_{l}} \right)$ (4)

$V_{l}=\frac{Lh^{2}}{4}\left( \theta-\frac{\pi}{2}+\sin\theta\cos\theta\right)({\cos^{2} \theta)}^{-1}$ (5)

The area of solid–liquid is constant, and the pressure at meniscus can be modified in Eq. (6)

${\Delta P}_{meniscus}=\frac{dU_{t}}{dV_{l}}=\gamma_{lg}\left( \frac{{dA}_{lg}}{dV_{l}} \right)=\gamma_{lg}\frac{2\left( 2\theta-\pi+2\sin\theta\cos\theta\right)}{h(2+\left( 2\theta-\pi\right)\tan\theta}$ (6)

When the COL1 was filling in ECM channel, filling flow was can be calculated by Poiseuille equation **(Eq. 7)** where *L*, *w,* and *h* were length, width and height of ECM channel, respectively, and *Q* is the flow rate of COL1 ^7^.

$P_{1,max}-P_{2}=\frac{12Q\mu L}{wh^{3}}\left[ 1-\sum_{n,odd}^{\infty} \frac{1}{n^{5}}\frac{192}{\pi^{5}}\frac{h}{w}\tanh\left( \frac{n\pi w}{2h} \right) \right]^{-1}$ (7)

At the end of COL1 filling, the pressure difference, *P_2_-P_air_*, is calculated by Young-Laplace equation and contact angle *θ_c_* was assumed to be 180°.

$P_{2}-P_{air}=-\frac{2\gamma(\cos\theta_{c})}{h}=\frac{2\gamma}{h}$ (8)

The pressure difference between the maximum pressure in liquid and air can be described by the following equation.

$P_{1,max}-P_{air}=\frac{2\gamma}{h}+\frac{12Q\mu L}{wh^{3}}\left[ 1-\sum_{n,odd}^{\infty} \frac{1}{n^{5}}\frac{192}{\pi^{5}}\frac{h}{w}\tanh\left( \frac{n\pi w}{2h} \right) \right]^{-1}$(9)


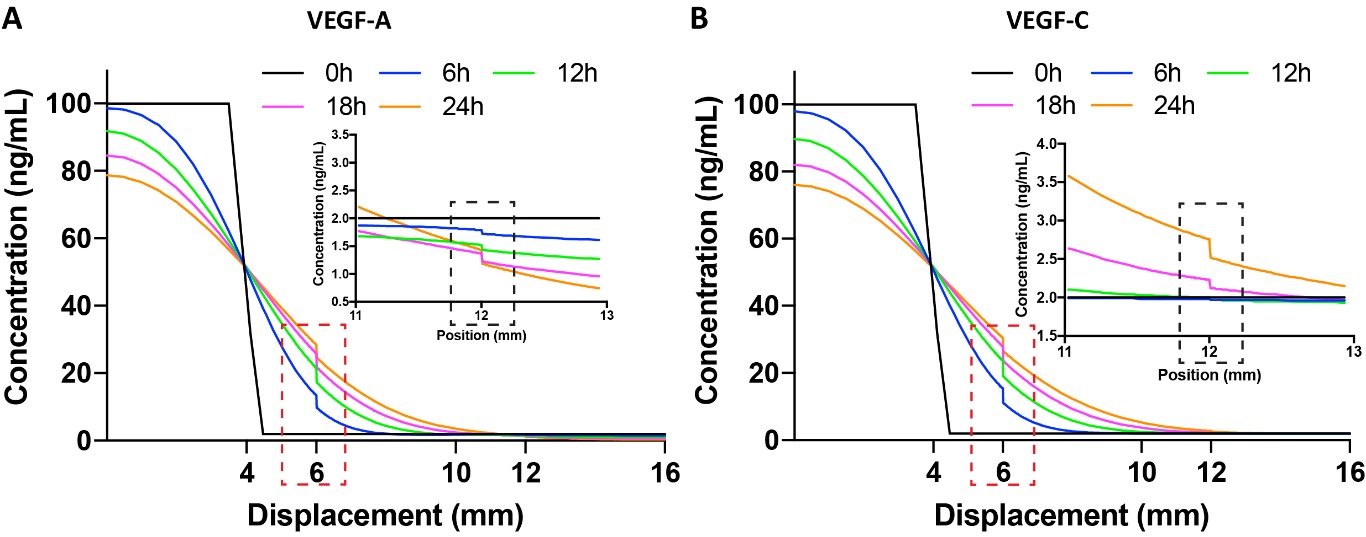


**Figure S6.** Computational simulation of growth factors, (a) VEGF-A and (b) VEGF-C. Molecular distribution in whole channels device, VEGF-A and -C, respectively.


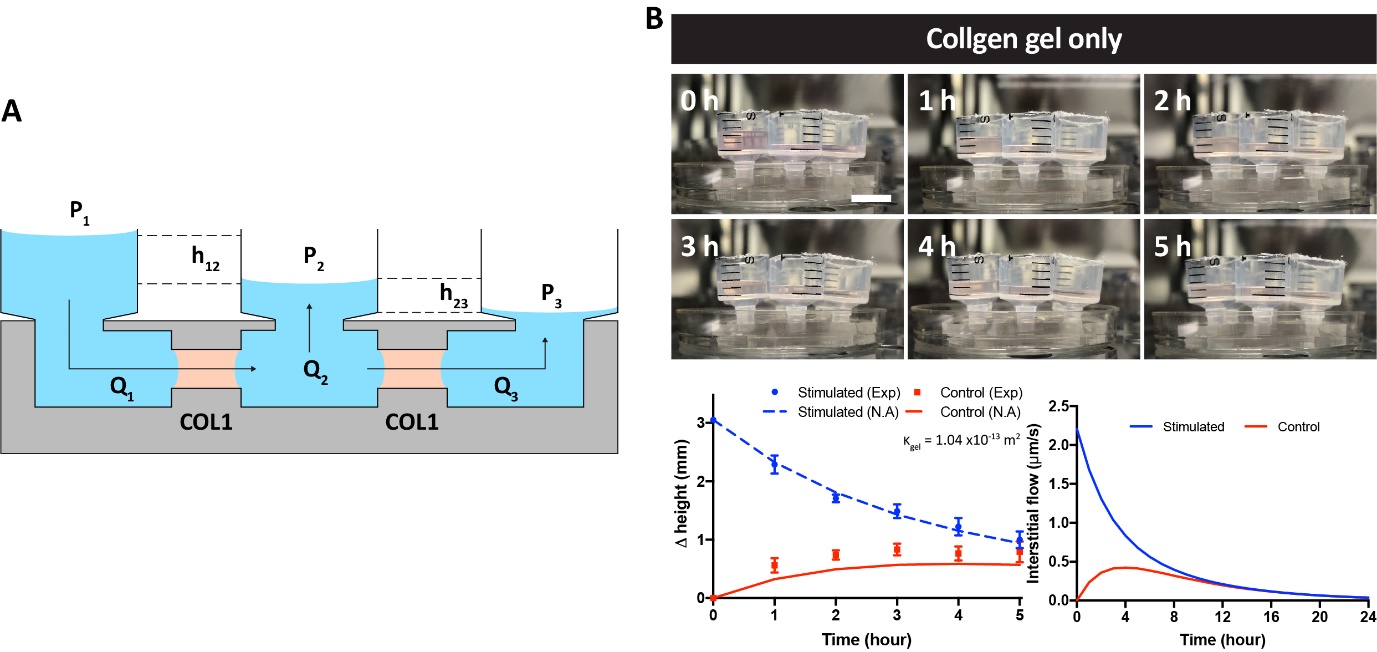


**Figure S7.** (a) Schematic illustration of interstitial flow generation. (b) Measurement Darcy permeability and calculation of generated interstitial flow in case of only collagen gel. Scale bar is 1 mm

For calculating interstitial flow, Darcy’s law (**Eq. 10**) and a continuity equation (**Eq. 11**) were used for the governing equation, where *Q*, *ΔP*, *A,* and *L* were the flow rate, pressure drop of the fluid, interface area between the ECM and medium channel and the width of the ECM channel, respectively. In addition, *κ* and *μ* indicate the permeability of a porous medium and the dynamic viscosity of the fluid, respectively.

$Q\left( t \right)= \frac{\kappa A\Delta P\left( t \right)}{\mu L}$ (10)

$Q_{1}\left( t \right)=Q_{2}\left( t \right)+Q_{3}\left( t \right)$ (11)

The pressure difference in each reservoir was calculated with height of the medium in a syringe.

$\Delta P_{ij}\left( t \right)=\rho gh_{ij}\left( t \right)$ (12)

$h_{12}\left( t \right)= \left\{ h_{12}\left( 0 \right)-\int\frac{Q_{1}\left( t \right)}{A_{r}}dt- \int\frac{Q_{2}\left( t \right)}{A_{r}}dt \right\}$ (13)

$h_{23}\left( t \right)= \left\{ h_{23}\left( 0 \right)+\int\frac{Q_{2}\left( t \right)}{A_{r}}dt- \int\frac{Q_{3}\left( t \right)}{A_{r}}dt \right\}$ (14)

Using Eqs. (10)–(12), flow rates of each component were derived by following equation.

$Q_{1}\left( t \right)= \frac{1}{2}\cdot\frac{\kappa\rho gA}{\mu L}\left\{ h_{12}\left( 0 \right)\cdot e^{-\frac{\kappa\rho gA}{\mu LA_{r}}t} +\left( h_{12}\left( 0 \right)-h_{23}\left( 0 \right) \right)\cdot e^{-\frac{3\kappa\rho gA}{\mu LA_{r}}t} \right\}$ (15)

$Q_{2}\left( t \right)= \frac{\kappa\rho gA}{\mu L}\left\{ h_{12}\left( 0 \right)-h_{23}\left( 0 \right) \right\} \cdot e^{-\frac{3\kappa\rho gA}{\mu LA_{r}}t}$ (16)

$Q_{3}\left( t \right)= \frac{1}{2}\cdot\frac{\kappa\rho gA}{\mu L}\left\{ h_{12}\left( 0 \right)\cdot e^{-\frac{\kappa\rho gA}{\mu LA_{r}}t}-\left( h_{12}\left( 0 \right)-h_{23}\left( 0 \right) \right)\cdot e^{-\frac{3\kappa\rho gA}{\mu LA_{r}}t} \right\}$ (17)

The interstitial flow was calculated by MATLAB


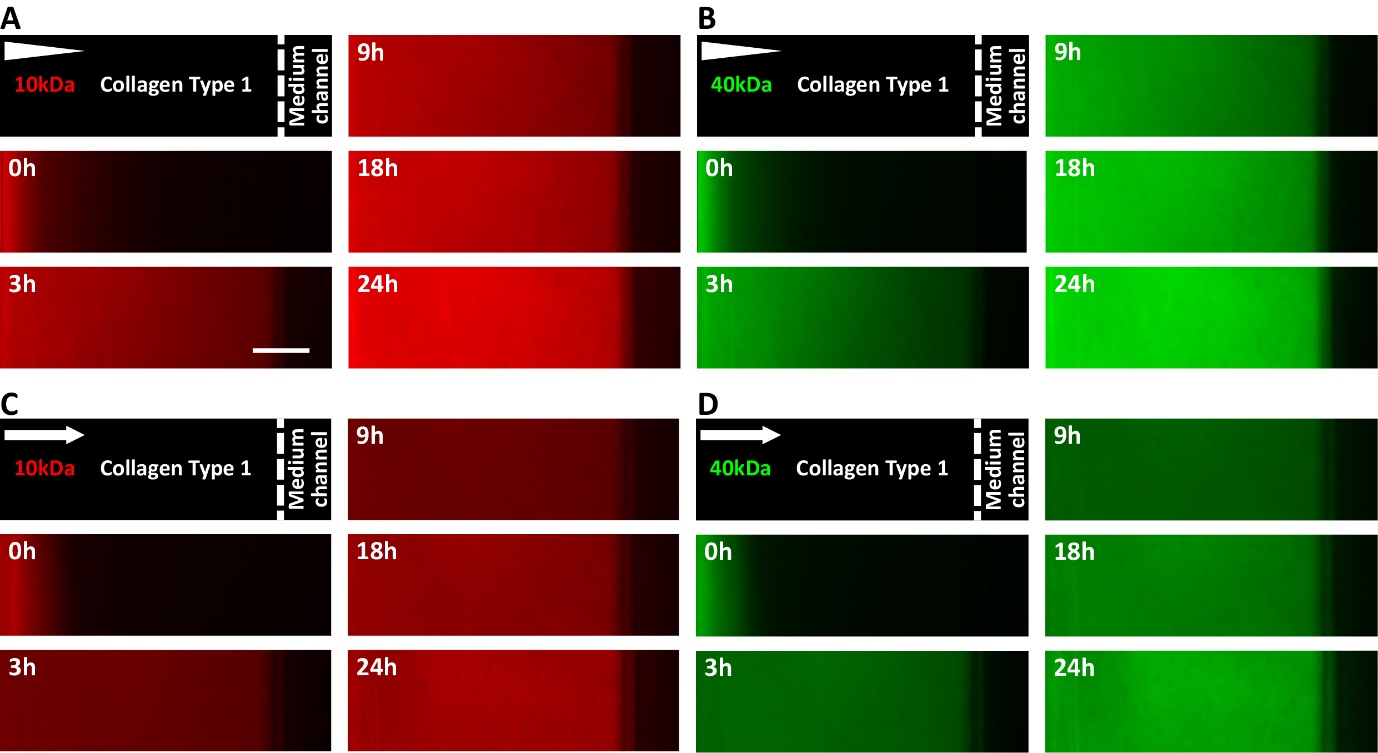


**Figure S8**. Fluorescent image of molecular distribution in COL1 under (a and b) no interstitial flow and (c and d) interstitial flow. (a, c) and (b, d) Experiments using 10kDa RITC-dextran and 10kDa FITC-dextran, respectively. Scale bar is 200 μm


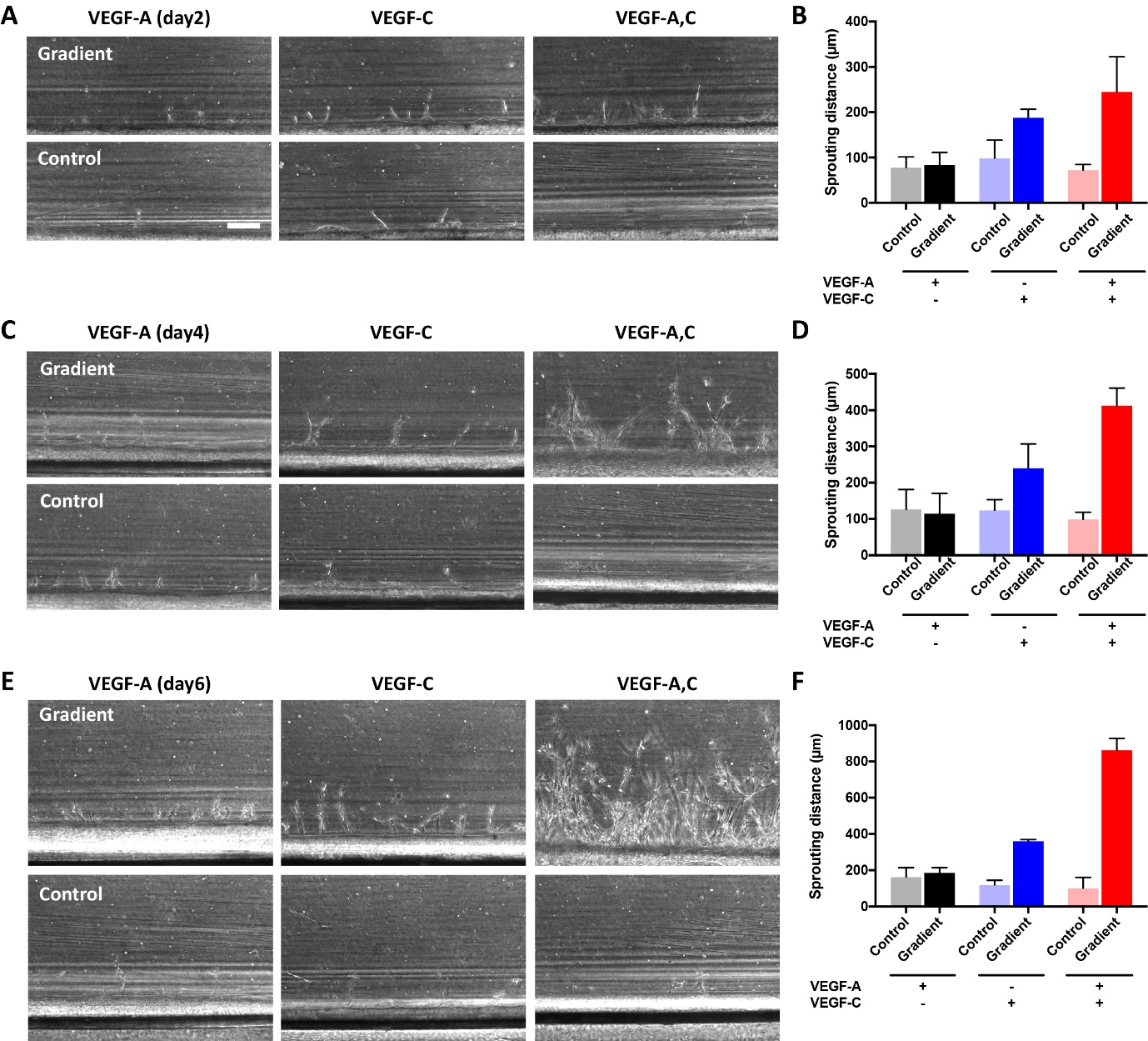


**Figure S9**. Phase-contrast image of lymphatic sprouting under various VEGF simulations in meso- fluidic device for (a) 2 days, (c) 4 days, and (e) 6 days, respectively. Scale bar is 200 μm. Sprouting distance of lymphatic endothelial cell for (b) 2 days, (d) 4 days, and (f) 6 days, respectively, and comparison with stimulated and control channel. Scale bar is 200 μm. (n = 4 and error bar indicates standard error).


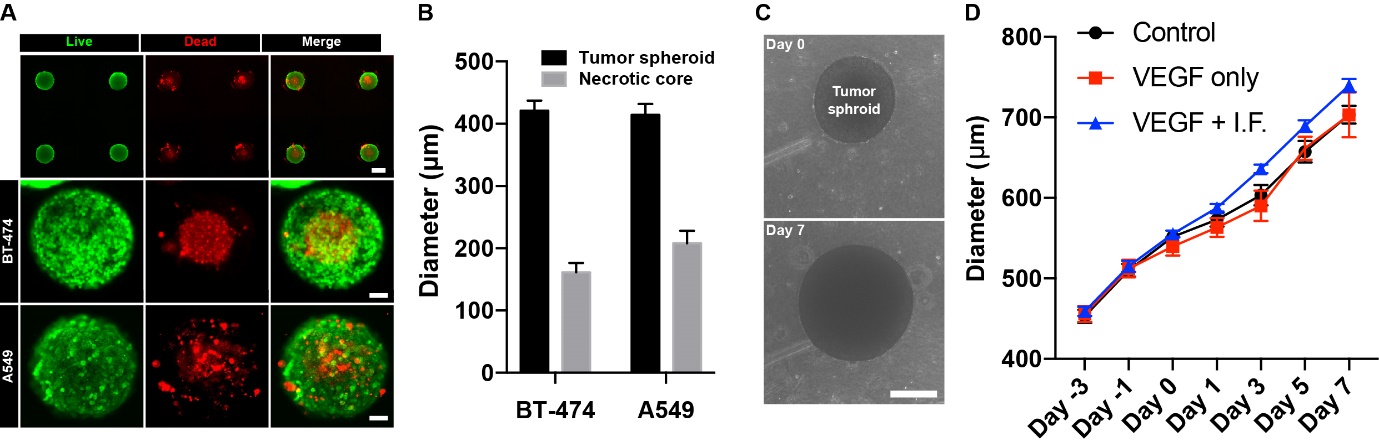


**Figure S10**. Tumor spheroid generation in concave well and cultured in macro fluidic device. (a) Live/dead staining using calcian-AM (green) and ethidium homodimer-1 (red). Scale bars are 200 μm, 100 μm, and 100 μm from upper row. (b) tumor spheroid size and necrotic core size in two types of cancer cell line (BT-474 and A549) (n = 5 and error bar indicates standard error). (c) Phase-contrast image of tumor spheroid in COL1 at day 0 and day 7. Scale bar is 300 μm. (d) Tumor spheroid growth measurement, stimulating lymphangiogenic factors.


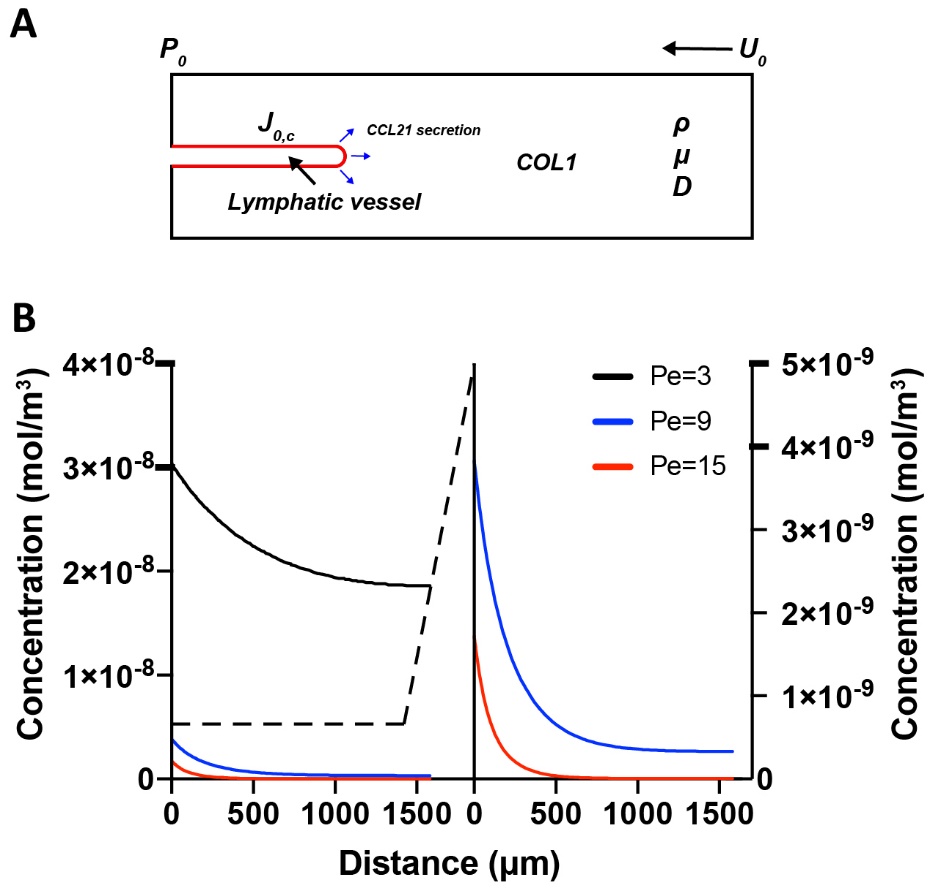


**Figure S11**. Computational simulation of CCL21 transport following various Péclet numbers, 3, 9, and 15. (a) Modeling of molecular transport for computational simulation. *P* and *U* indicate fluid pressure (Pa) and initial velocity (m/s) in COL1, respectively. In addition, *ρ, μ, D*, and *J* indicate medium density (kg/m^3^), dynamic viscosity (Pa⋅s), diffusion coefficient (m^2^/s), and molecular flux (mol/m^2^⋅s), respectively. (b) Simulation results of concentration distribution in COL1.
